# LncRNA PRKCA-AS1 promotes LUAD progression and function as a ceRNA to regulate S100A16 by sponging miR-508-5p

**DOI:** 10.7150/jca.91184

**Published:** 2024-01-27

**Authors:** Chaohui Wu, Jiansheng Yang, Xianbin Lin, Jingyang Wu, Chuangcai Yang, Shuchen Chen

**Affiliations:** 1 Department of Thoracic and Cardiovascular Surgery, The Second Affiliated Hospital of Fujian Medical University, Quanzhou, Fujian, 362000, China.; 2 Department of Thoracic Surgery, Fujian Medical University Union Hospital, Fuzhou, Fujian, 350001, China.

**Keywords:** lung adenocarcinoma, LncRNA PRKCA-AS1, miR-508-5p, S100A16, ceRNA

## Abstract

**Objective:** This study aimed to elucidate the underlying mechanism of LncRNA PRKCA-AS1 in lung adenocarcinoma (LUAD).

**Methods:** The expression of LncRNA PRKCA-AS1, miR-508-5p and S100A16, in LUAD tissues or cell lines (NCI-H520 and H1299) was analyzed with qRT-PCR. The clinical diagnostic value of LncRNA PRKCAAS1, miR-508-5p and S100A16 in LUAD were analyzed by receptor operating characteristic (ROC) curve. Then we knockdown or overexpression of PRKCAAS1 in NCI-H520 and H1299 cells, and the cell function test was applied to detect the activity and metastasis level of cells in different transfection groups. Then Pearson correlation analysis was used for the correlation between miR-508-5p and PRKCA-AS1. The dual-luciferase reporter experiment and CHIRP analysis was conducted to verify the target binding relationship of PRKCA-AS1, miR-508-5p or S100A16. FISH assay analyzed the colocalization of PRKCA-AS1 and miR-508-5p in NCI-H520 and H1299 cells. Rescue experiment and tumorigenesis experiment in nude mice further explore the regulatory mechanisms of LncRNA PRKCA-AS1, miR-508-5p and S100A16 on LUAD progression in vitro and in vivo.

**Results:** From the results, PRKCA-AS1 and S100A16 were up-regulated in LUAD tissues, while miR-508-5p was downregulated compared with the adjacent tissues. And gain-of-function revealed that PRKCA-AS1 knock-down apparently suppressed the cell proliferation and metastasis, whereas miR-508-5p inhibitors or S100A16 overexpression showed a opposite effect. In addition, there is evidence that PRKCA-AS1, miR-508-5p and S100A16 have a targeted regulatory relationship. Moreover, rescue experiment and tumorigenesis experiment in nude mice further confirmed that LncRNA PRKCA-AS1 regulates S100A16 through sponging miR-508-5p to regulate LUAD progression in vitro and in vivo.

**Conclusion:** These results demonstrated that LncRNA PRKCA-AS1 might regulate LUAD by acting as a ceRNA via sponging miR-508-5p and regulating S100A16 expression, indicating that manipulation of PRKCA-AS1 might be a potential therapeutic strategy in LUAD.

## 1. Introduction

Lung adenocarcinoma (LUAD) is an important pathological type of non-small cell lung cancer (NSCLC), accounting for about 45% of lung cancer and increasing year by year, especially in young people 1. In recent years, the progress has been made in lung cancer research, but the overall survival rate with an average 5-year survival rate of less than 20% is obviously unsatisfactory 2. The inadequate understanding of the biological mechanism of LUAD is the main reason that limits the improvement of the therapeutic effect. Consequently, it is imminently required to further explore novel early diagnosis and clarify the pathogenesis of tumor, so as to improve the prognosis for LUAD.

Long non-coding RNAs (lncRNAs), a class of noncoding RNA transcripts with exceeding 200 nucleotides in sequence, have been proved to exhibit extensive biological functions, such as chromatin remodeling, transcription, splicing, translation, and epigenetic regulation, through cross-talk with other RNA species and via multiple chromatin-based mechanisms 3, 4. Accumulating studies indicated that dysfunction or alteration of lncRNAs could lead to the aberrant expression of related genes that promotes tumor formation and progression of various cancer types 5-7. In 2011, the competitive endogenous RNA (ceRNA) hypothesis was proposed for the first time, which believed that lncRNA or circRNA could regulate mRNA expression by competing with mRNA for common binding sites on target miRNAs 8. Subsequently, the roles of lncRNAs in regulating tumor progression have been largely published in many cancers 9-11, including lung cancer 12, 13. Herein, we explored the downstream mechanism of lncRNA PRKCA-AS1 as a ceRNA to regulate tumor progression in LUAD, which has not been explored in tumor so far.

S100 protein family is a calcium binding protein, which includes 21 known members 14, who participate in the basic cellular processes by participating in a variety of different ways 15. More importantly, the role of S100 proteins in tumors deserves further attention 16. Researches showed that the function of S100 proteins in malignant melanoma 17, pancreatic cancer 18 and lung cancer 19 has been studied to a certain extent. In addition, S100 families have been found to be related to the pathogenesis and invasion of low-grade glioma, S100A2, S100A6, S100A10, S100A11, S100A16, S100A1 and S100A13 were differential expression in low-grade glioma 20. There was evidence that S100A16, as an oncogene, can play a role in pancreatic cancer through FGF19 mediated AKT pathway 21. In addition, various evidences have indicated that S100A16 regulates AKT signal transduction in cervical cancer (HeLa cells) 22, bladder cancer (M-RT4 cells) 23, prostate cancer (DU-145 and PC-3 cells), etc., thus affecting cancer progression. However, S100A16/AKT signal transduction has not been reported in the study of lung cancer.

## 2. Methods

### 2.1 Tissue specimens, cell lines, and transfection

LUAD and matched adjacent non-tumor tissue samples were obtained from 30 patients in The Second Affiliated Hospital of Fujian Medical University from September 2021 to February 2022 were included, and the pathological diagnosis of these patients was LUAD. All specimens were subjected to qRT-PCR for PRKCA-AS1, miR-508-5p and S100A16 detection.

The cell lines NCI-H520 and H1299 were obtained from the American Type Culture Collection (ATCC) and cultured in Dulbecco's Modified Eagle Medium (DMEM) supplemented with 10% fetal bovine serum (FBS, Gibco, USA). The culture conditions were maintained at 37°C with 5% CO2 in a humidified atmosphere.

To investigate the function of LncRNA PRKCA-AS1, gene silencing and overexpression experiments were conducted on these two cell lines. Transfection was performed using Lipofectamine 3000 (Invitrogen, CA) following the manufacturer's protocol. Specifically, cells were seeded in 6-well plates one day prior to transfection to ensure that cell density reached about 70-80% at the time of transfection. The transfection mixture included appropriate amounts of PRKCA-AS1, miR-508-5p inhibitor, S100A16 expression plasmid, and corresponding control plasmids. After transfection, the cells were further cultured under normal conditions for 48 hours for subsequent experimental analyses.

This study was approved by the Ethics Committee of The Second Affiliated Hospital of Fujian Medical University.

### 2.2 qRT-PCR

For the mRNA qualification, total RNA in LUAD tissues or NCI-H520 and H1299 cells was extracted with TRIzol solution (Invitrogen, USA). Then total RNA was transcribed into cDNA with PrimeScript RT reagent kit (TaKaRa, Japan). And the level of PRKCA-AS1, miR-508-5p and S100A16 were detected by employing Prime Script^TM^ RT Master Mix Kit (Takara, Japan). β-actin and U6 were served as internal control.

The primers for amplification were as follows:

LncRNA-PRKCA-AS1, F: 5'-ACTAACCCAAACCCAGAACAC-3' and R: 5'-CTTGAAATTACACAGCCAGGAATG-3';

hsa-miR-508-5p, F: 5'-CGTACTCCAGAGGGCGTCA-3' and R: 5'-AGTGCAGGGTCCGAGGTATT-3';

S100A16, F: 5'-TGCTCCAGAAAGAGCTGAACC-3' and R: 5'-CCGCCTATCAAGGTCCAGTA-3';

U6, F: 5'-CAAATTCGTGAAGCGTT-3' and R: 5'- TGGTGTCGTGGAGTCG-3';

β-actin, F: 5'-TGACGTGGACATCCGCAAAG-3' and R: 5'-CTGGAAGGTGGACAGCGAGG-3'.

### 2.3 CCK8 assay

The cell viability of LUAD cells was detected with Cell Counting Kit-8 (CCK-8, Dojindo, Japan). First, 5×10^3^ cells/well was suspended and cultured for 0 to 72 h. After that, 10 µL of CCK-8 solution was added, and MRX II microplate reader (Dynex Technologies, USA) was used to examine the absorbance of each well at 450 nm.

### 2.4 Clone formation assay

NCI-H520 and H1299 cells of different groups were inoculated into culture dishes (60 mm), incubated with medium containing 10% FBS, and then cultured in 5% CO2 at 37 ℃. About 3 weeks later, visible colonies appeared in the culture dish, and they were fixed and stained. Then record the number of colonies and calculate the colony formation rate.

### 2.5 Transwell assays

For the migration analysis, NCI-H520 and H1299 cells (5 × 10^4^) were seeded into upper transwell chambers with 8-μm pores (Corning, USA). The culture medium in the device is DMEM culture medium, the difference is that 1% FBS is added in the upper chamber and 15% FBS is supplemented in the lower chamber. And for the invasion assay, Matrigel matrix was added in the upper transwell chambers. After 48h of culture, the cells were fixed and stained, and then the number of cells in the chamber was counted.

### 2.5 Dual-luciferase reporter assay

The dual-luciferase reporter experiment was carried out to verify the target binding relationship of PRKCA-AS1, miR-508-5p and S100A16. The putative binding site with the mutant sequence of (PRKCA-AS1-MUT, S100A16-MUT) and the wild-type sequence (PRKCA-AS1-WT, S100A16-WT) were cloned into the psiCHECK-2 vector, respectively. And then co-transfecting the cells with 50 nM miR-508-5p mimics or inhibitors and 0.5 mg reporter plasmid. After 48 h, the Dual-Luciferase Reporter Assay System (Promega, USA) was employed for the luciferase detection.

### 2.6 Western blot

NCI-H520 and H1299 cells were first lysed with RIPA lysis buffer, then the total protein concentration was evaluated with the protein assay kit (Beyotime Biotech, China). Total protein concentration was probed with primary antibodies : S100A16 (1: 1000, K008596P, Solarbio), AKT (1: 1000, 10176-2-AP, Proteintech), p-AKT (1: 2000, 66444-1-Ig, Proteintech), N-cadherin (1: 1000, AB18203, abcam), Vimentin (1: 2000, K008105P, Solarbio), E-cadherin (1: 5000, 20874-1-ap, Proteintech), and β-actin (1: 5000, ab8226, abcam), followed by detection with an ECL kit (Thermo Scientific, USA). Finally, the protein was quantitatively analyzed using chemidoc imaging system (Tanon, China) and ImageJ analysis software.

### 2.7 Subcellular fractionation and RNA fluorescence in situ hybridization (FISH) assays

The FISH probes for PRKCA-AS1, miR-508-5p, S100A16 and U6 were synthesized and produced (GenePharma, China). An RNA FISH kit (Genepharma, China) was used for the expression and localization of PRKCA-AS1, miR-508-5p and S100A16 in NCI-H520 and H1299 cells, and the probe sequences are listed in Supplementary Table 1. we first seeded the cells in climbing slides, the cells were fixed with 4% paraformaldehyde, then the FISH assay was conducted. At last, the fluorescence images were captured with confocal microscope. The FISH probes were listed as follows:

PRKCA-AS1: 5'-DIG-taaaaccaaacggccagatttctaagcg

S100A16: 5'-DIG-atatttgtagaagttttccaccaggaca

miR-508-5p: Catgagtgacgccctctggagta

### 2.8 Chromatin isolation by RNA purification (ChIRP) assay

The ChIRP assay was conducted with the ChIRP assay kit (Guangzhou, China) and the biotinylated PRKCA-AS1 probe sequences are shown in Supplementary Table 1. As described previously 24, H1299 cells were used and crosslinked in glutaraldehyde, and quenched with glycine. Then the cells were lysed in ChIRP lysis buffer at 4 °C, and hybridized with probes for 4 h in a hybridization oven.

The CHIRP biomarker probes were listed as follows:

1, Aagtatcaagcttctctggc-bio;

2, Tcttgacatgagcttcaacc-bio;

3, Tttcctgactgactgaaggg-bio;

4, gagtacgacttccaacatgt-bio.

### 2.9 Mouse tumor models

BALB/c nude mice were provided by a professional experimental animal supply center. All animal experiments were approved by the Ethics Committee of The Second Affiliated Hospital of Fujian Medical University and were conducted in strict accordance with relevant ethical guidelines for animal experimentation.

The nude mice were housed under specific pathogen-free (SPF) conditions to minimize the risk of infection. The housing environment was maintained at a temperature of 22-24℃ and a humidity of 50-60%, with adequate ventilation. The mice were kept in standard cages, with 4-5 mice per cage. The environment provided ample daylight to simulate natural light-dark cycles. The diet of the nude mice was managed by professional animal caretakers, providing standard sterile rodent feed and clean drinking water. To ensure the cleanliness of the housing environment and the health of the animals, cages were regularly cleaned, and food and water were replaced. In addition, regular health checks were performed on the mice to ensure they were free from infections and other health problems.

During the experiments, standard operating procedures were followed to minimize discomfort to the animals. All surgeries and treatments were performed under anesthesia to alleviate pain. At the end of the experiments, the animals were humanely euthanized by CO2 asphyxiation, followed by the collection of tissue samples.

### 2.10 H&E staining

Histopathological examination of LUAD tissues was performed with HE staining. In short, the paraffin sections were dewaxed first, and then hematoxylin staining and differentiation were performed. Put the slices in hematoxylin for about 5 minutes, rinse with distilled water, then put the slices in eosin for staining for 2 minutes, and finally dehydrate the slices. Finally, the sections were observed with a microscope.

### 2.11 Statistical analysis

All the data were analyzed with Prism 8.0 (GraphPad, USA), presented as mean ± SD. For differences comparisons between groups, unpaired Student's *t* test or one-way analysis of variance (ANOVA) was employed. *p* < 0.05 was indicated statistical significance.

## 3. Results

### 3.1 LncRNA PRKCA-AS1 is highly expressed in LUAD

**Figure 1:**
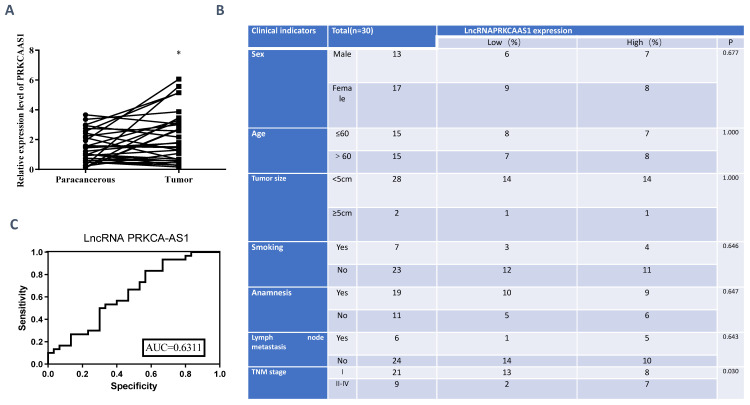
** LncRNA PRKCA-AS1 is highly expressed in LUAD.** (A) The expression of PRKCA-AS1 in LUAD and adjacent tissues was detected with qRT-PCR. (B) Information collected about 60 patients. (C) ROC curve analysis of clinical diagnostic significance of LncRNA PRKCAAS1. *, *p* < 0.05; **, *p* < 0.01; ***, *p* < 0.001.

To reveal the functions of LncRNA PRKCA-AS1 in LUAD, we first detected the expression of PRKCA-AS1 in LUAD and adjacent tissues. From the result, compared with the normal group, PRKCA-AS1 was up-regulated in LUAD (Figure 1A). The tumor node metastasis (TNM) classification displayed that high PRKCA-AS1 was markedly corresponded to an advanced tumor stage in LUAD patients (Figure 1B). ROC curve analysis showed that LncRNA PRKCAAS1 has certain clinical diagnostic significance (Figure 1C). These results suggested that LncRNA PRKCA-AS1 was up-regulated in LUAD and was associated with poor prognosis.

### 3.2 Differential expression of PRKCAAS1 affected LUAD Cell Proliferation and metastasis

**Figure 2:**
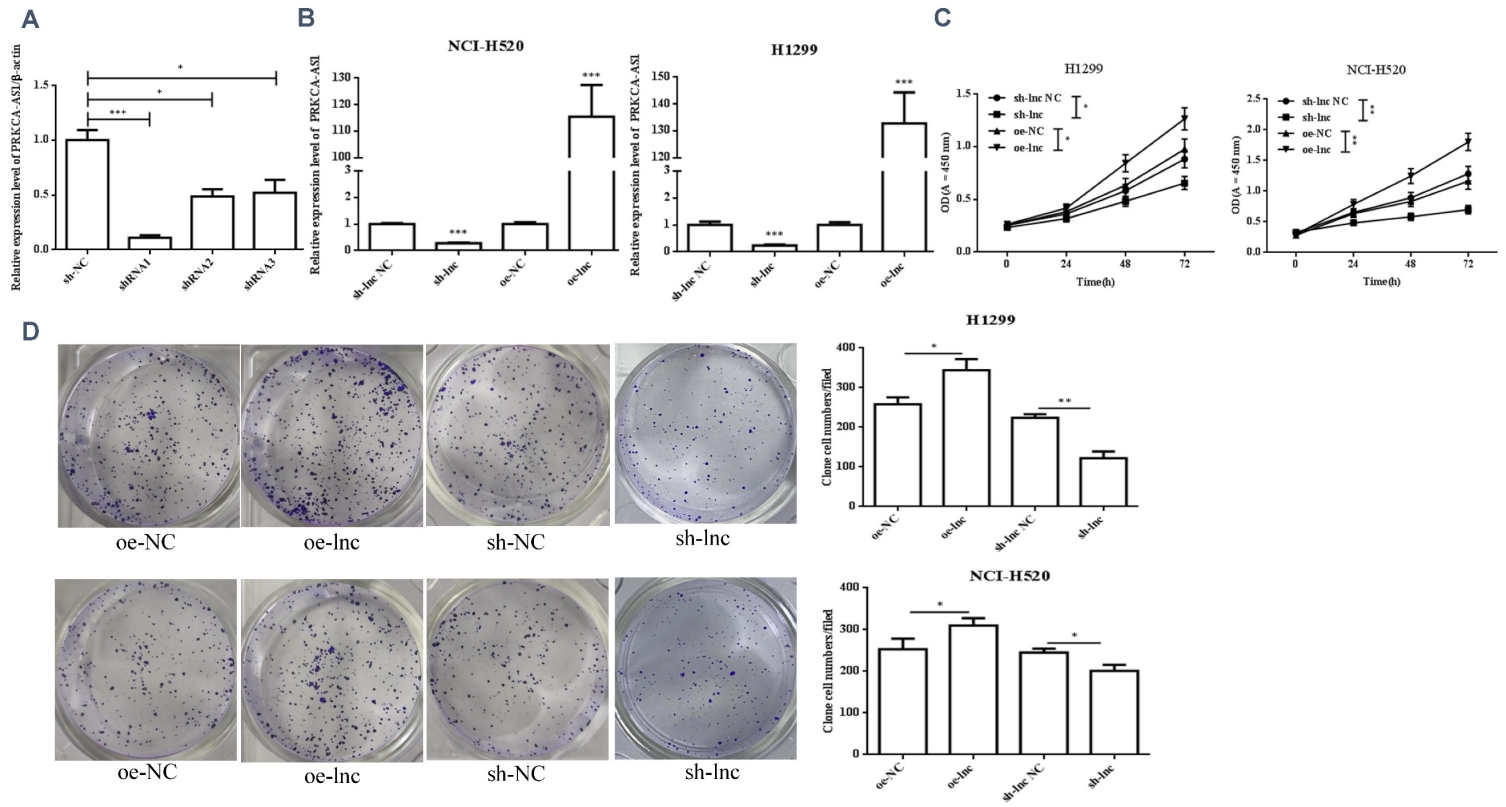

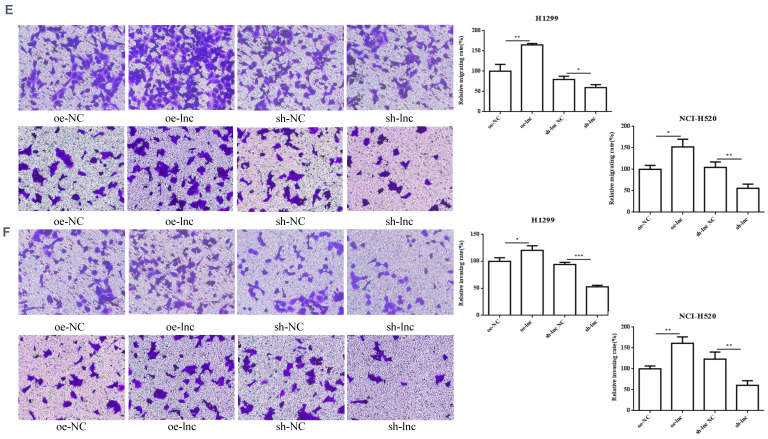
** Differential expression of PRKCAAS1 affected LUAD Cell Proliferation and metastasis.** (A-B) The transfection efficiency of PRKCAAS1 was confirmed by qRT-PCR. (C) CCK-8 assay was used to detect the vitality of NCI-H520 and H1299 cell. (D) Colony formation experiments was used for proliferation ability of NCI-H520 and H1299 cell. (E-F) The transwell assay analyzed the cell migration and invasion abilities in NCI-H520 and H1299 cells. *, *p* < 0.05; **, *p* < 0.01; ***, *p* < 0.001.

We interference or overexpression of PRKCAAS1 in NCI-H520 and H1299 cells. To prevent the effect of off-target, multiple interfere sequences are set up, and the transfection efficiency was confirmed by qRT-PCR (Figure 2A-B). CCK-8 and colony formation experiments revealed that PRKCAAS1 overexpression substantially increased NCI-H520 and H1299 cell proliferation abilities in vitro. On the contrary, low expression of PRKCAAS1 inhibited cell growth (Figure 2C-D). The transwell assay reported that upregulated of PRKCAAS1 improved cell migration and invasion abilities in NCI-H520 and H1299 cells, while PRKCAAS1 knockdown could inhibit cell metastasis (Figure 2E-F). These results verified that the differential expression of PRKCAAS1 could affect the proliferation and metastasis ability of LUAD in vitro.

### 3.3 MiR-508-5p was down-regulated in LUAD and negatively regulated by LncRNA PRKCA-AS1

**Figure 3:**
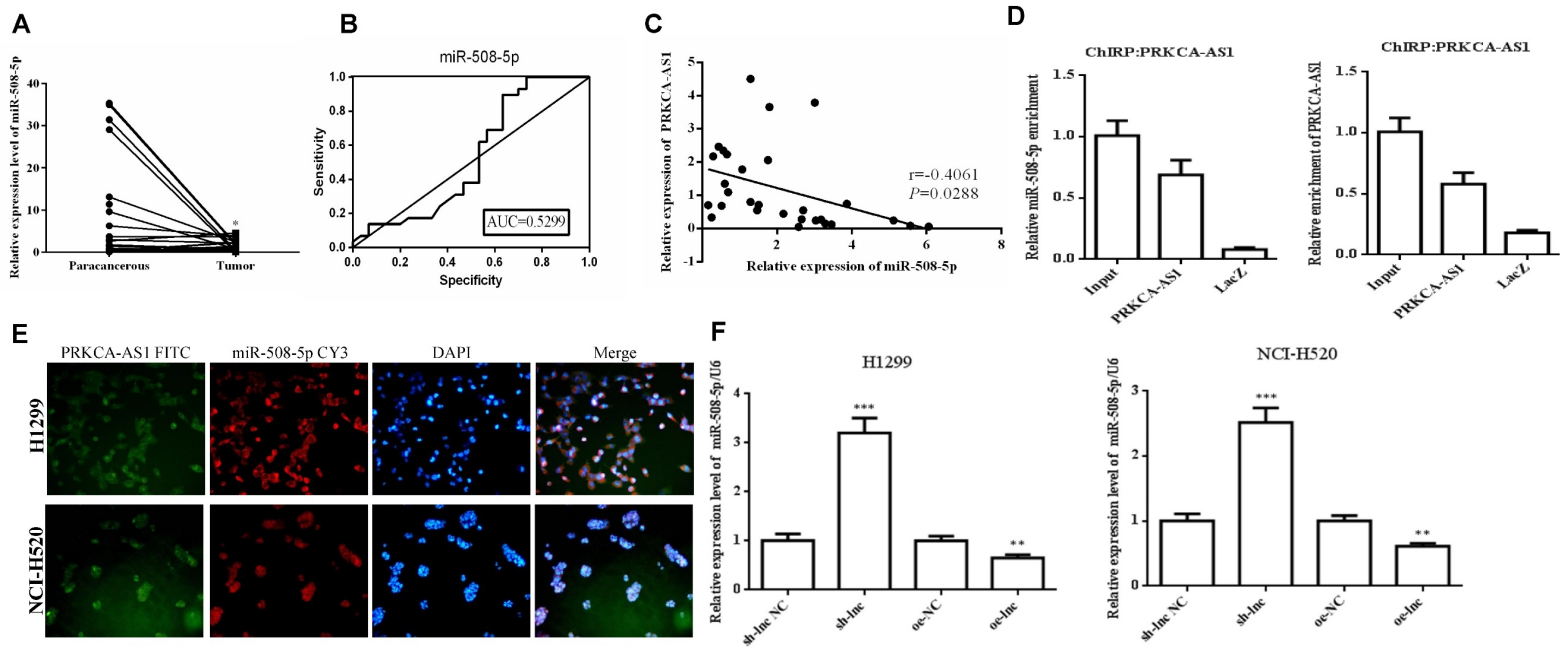
** MiR-508-5p was down-regulated in LUAD and negatively regulated by LncRNA PRKCA-AS1.** (A) The expression of miR-508-5p in LUAD and adjacent tissues was detected with qRT-PCR. (B) ROC curve analysis of clinical diagnostic significance of miR-508-5p. (C) Pearson Correlation analysis was used for the relationship of miR-508-5p and PRKCA-AS1. (D) The targeting relationship of PRKCA-AS1 and miR-508-5p was verified with CHIRP assay. (E) The location of PRKCA-AS1 and miR-508-5p was determined by FISH analysis. (F) The expression of miR-508-5p in different transfection groups was detected with qRT-PCR. *, *p* < 0.05; **, *p* < 0.01; ***, *p* < 0.001.

To further study the downstream molecular mechanism of PRKCAAS1 affecting the progress of LUAD, we screened the downstream miRNAs of PRKCAAS1, and miR-508-5p attracted our attention. Thus, the qRT-PCR and ROC curve analysis were conducted and the results revealed that miR-508-5p were substantially reduced in LUAD tissues, and has certain clinical diagnostic significance in LUAD patients (Figure 3A-B). Then Pearson Correlation analysis found that miR-508-5p expression was negatively affected by PRKCA-AS1 (Figure 3C). CHIRP assay confirmed that PRKCA-AS1 and miR-508-5p have a targeting relationship (Figure 3D). Further, in order to determine the location of PRKCA-AS1 and miR-508-5p, FISH analysis of NCI-H520 and H1299 cells showed that both were mainly located in the cytoplasm (Figure 3E). Additionally, si-PRKCA-AS1 could expedite miR-508-5p expression in NCI-H520 and H1299 cells, while oe-PRKCA-AS1 could obviously repress miR-508-5p (Figure 3F). The data above indicated that miR-508-5p was inhibited in LUAD and negatively regulated by LncRNA PRKCA-AS1.

### 3.4 LncRNA PRKCA-AS1 regulated S100A16 and AKT pathway by sponging miR-508-5p

**Figure 4:**
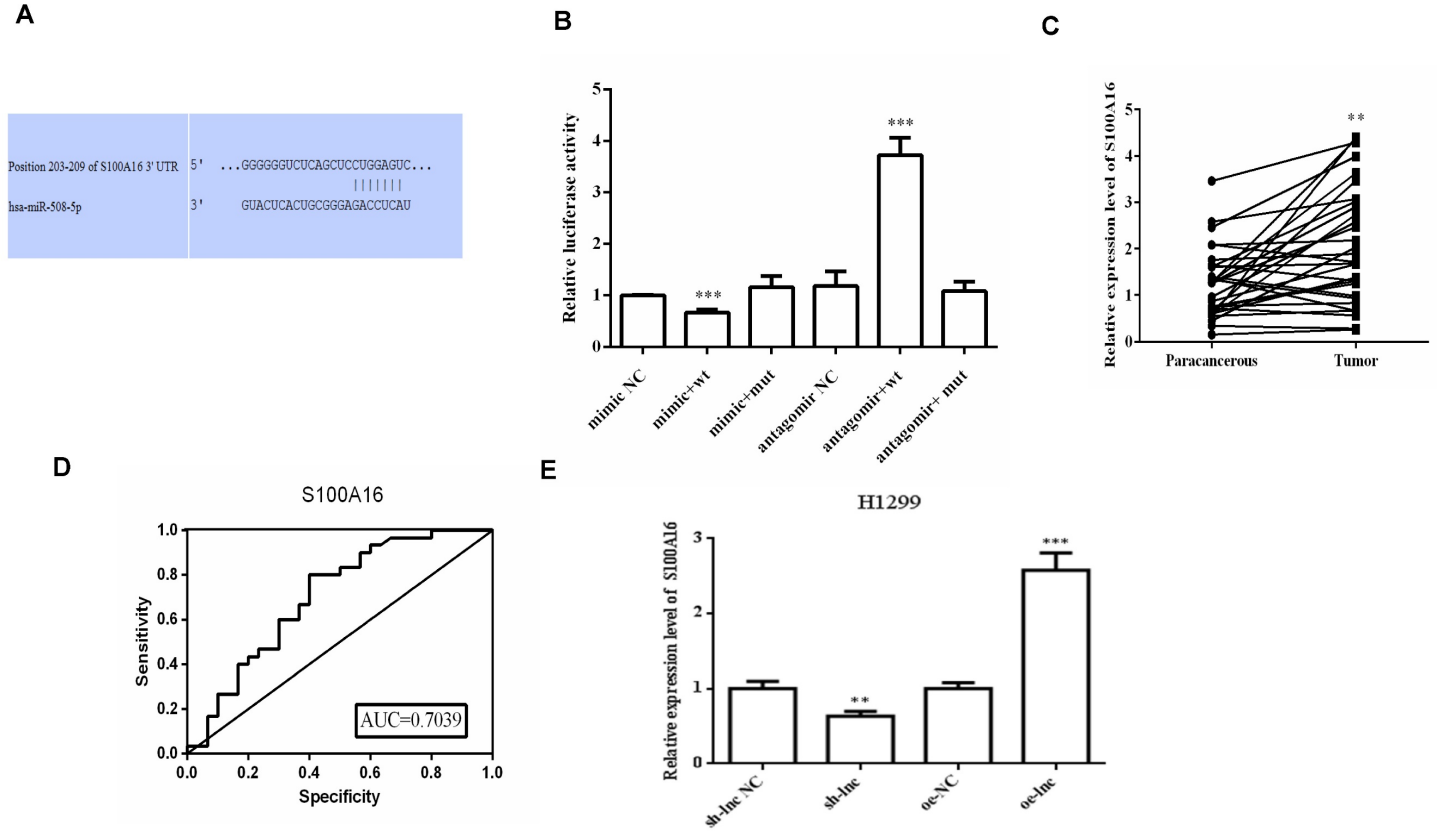

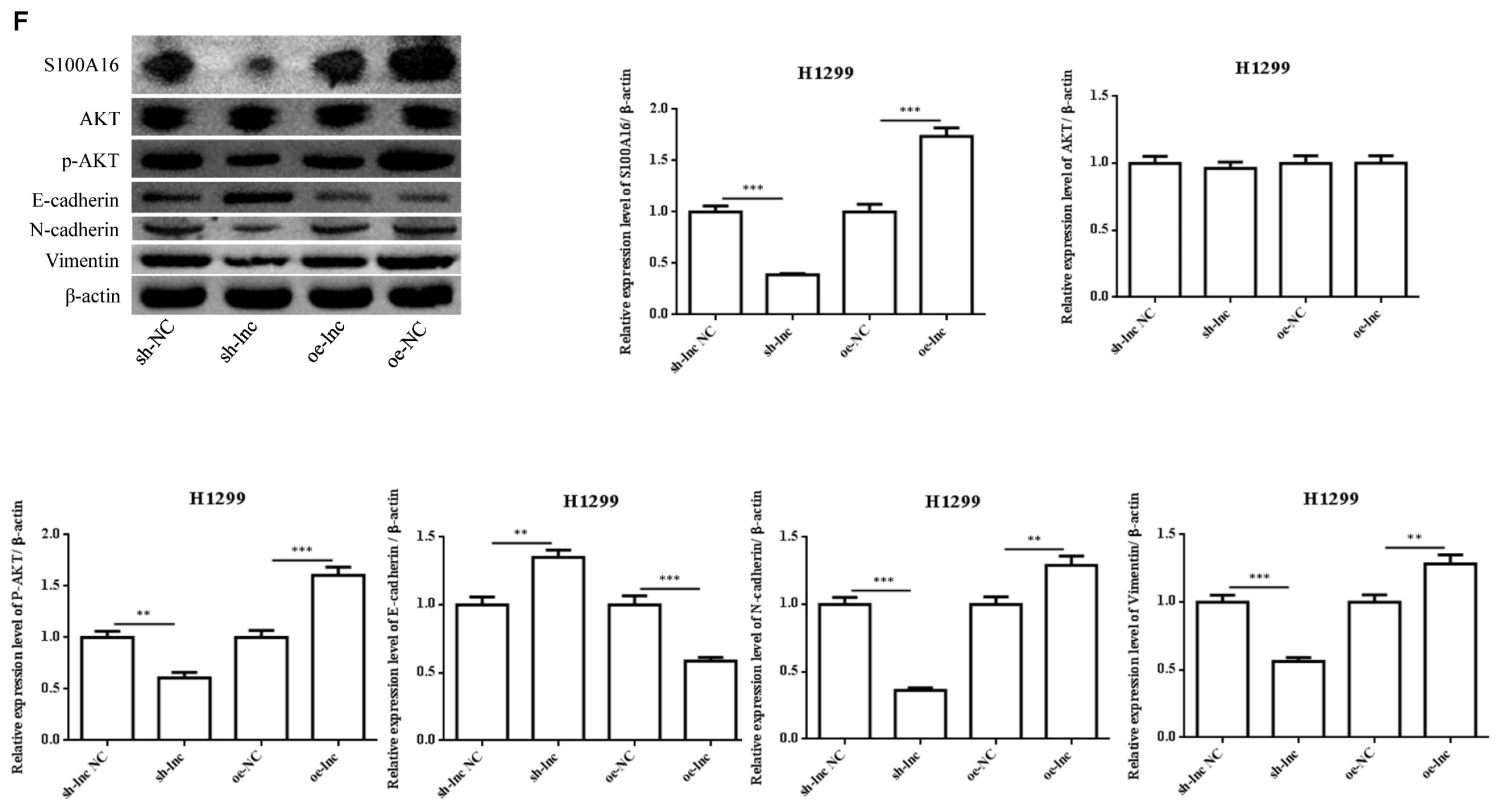
** LncRNA PRKCA-AS1 regulated S100A16 and AKT pathway by sponging miR-508-5p.** (A) Online websites predict the binding sites of miR-508-5p and S100A16. (B) Dual-luciferase reporter assay verified the binding relationship between the two. (C) The expression of S100A16 in LUAD and adjacent tissues was detected with qRT-PCR. (D) ROC curve analysis of clinical diagnostic significance of S100A16. (E) The mRNA level of S100A16 in different transfection cells was analyzed by qRT-PCR. (F) Western blot was used to detect the protein expression of S100A16, AKT, p-AKT, E-cadherin, N-cadherin, and Vimentin. *, *p* < 0.05; **, *p* < 0.01; ***, *p* < 0.001.

There was evidence that lncRNAs, acting as ceRNAs, could indirectly regulate mRNA expression by sponge miRNAs 9. We used online targetscan website and double luciferase reporter gene to predict and verify the relationships between miR-508-5p and S100A16 3'UTR (Figure 4A-B). Then qRT-PCR and ROC curve analysis proved that the level of S100A16 in LUAD tissues was apparently increased, which has certain clinical diagnostic significance for LUAD patients (Figure 4C-D). Moreover, PRKCA-AS1 knockdown markedly restrained the mRNA and protein expression of S100A16, p-AKT, N-cadherin and vimentin, but induced E-cadherin. On the other hand, oe-PRKCA-AS1 showed opposite results, but all groups did not affect AKT expression (Figure 4E-F).

### 3.5 LncRNA PRKCA-AS1 regulates LUAD progression by modulating S100A16/AKT via sponging miR-508-5p in vitro

**Figure 5:**
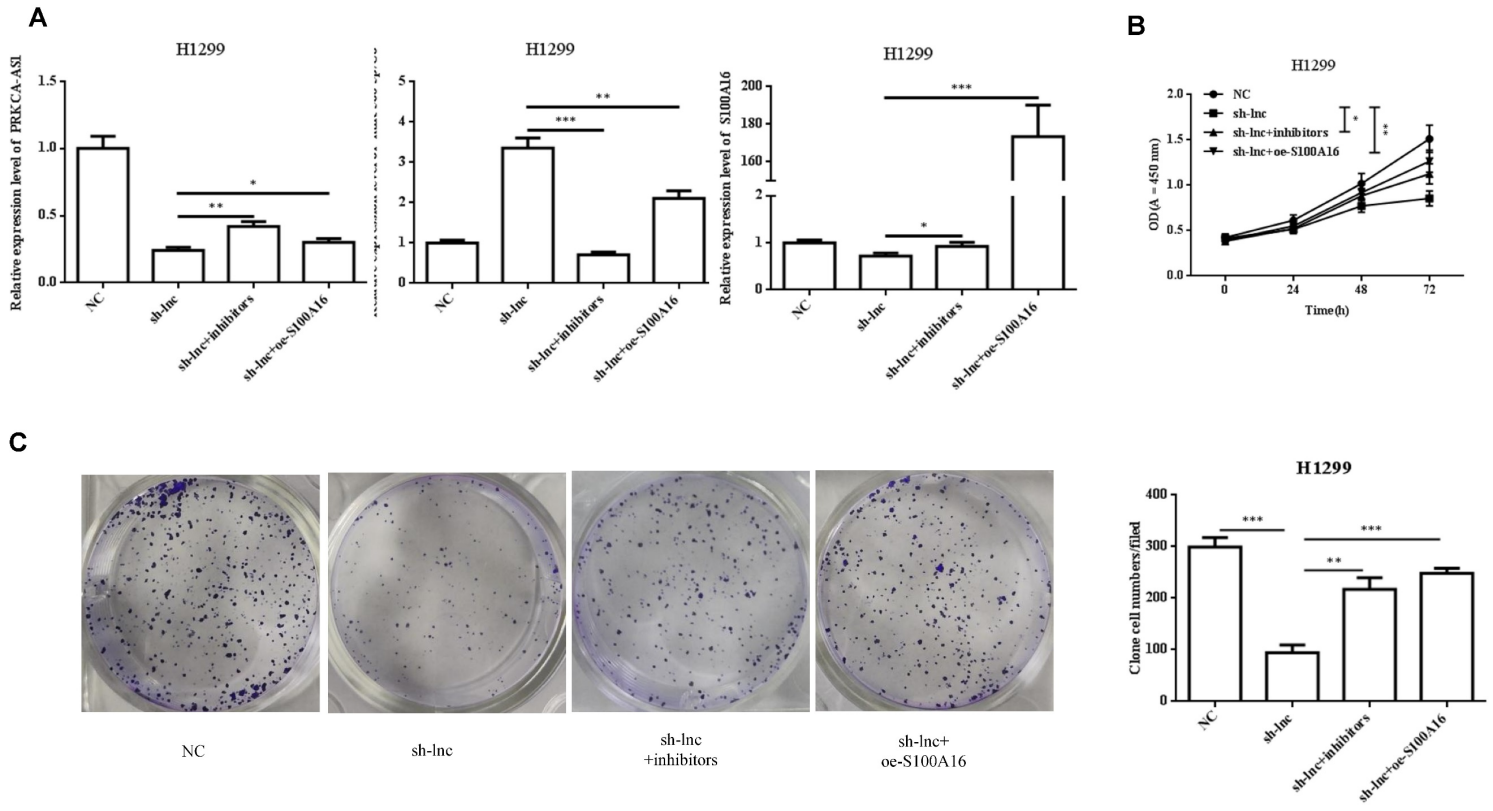

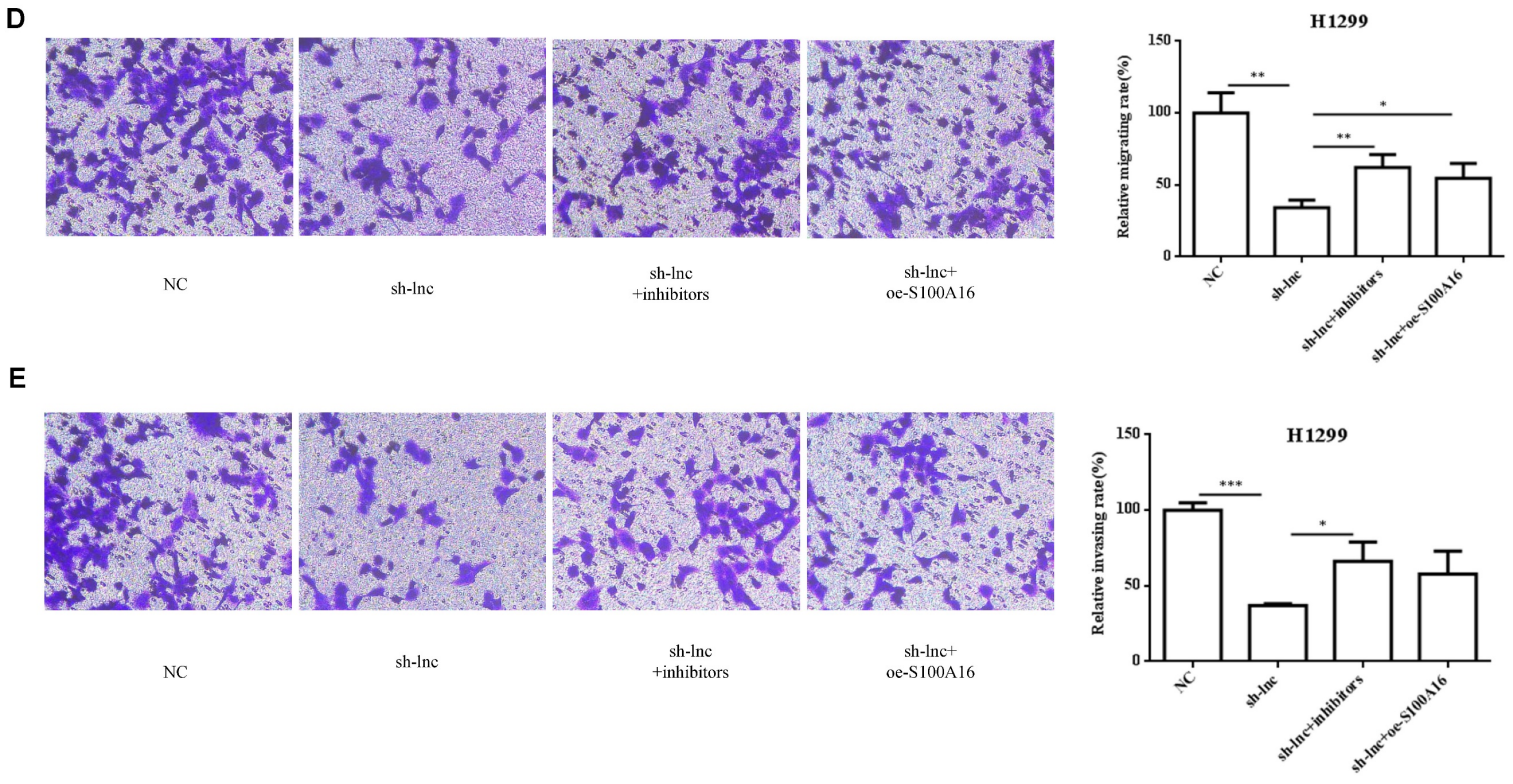

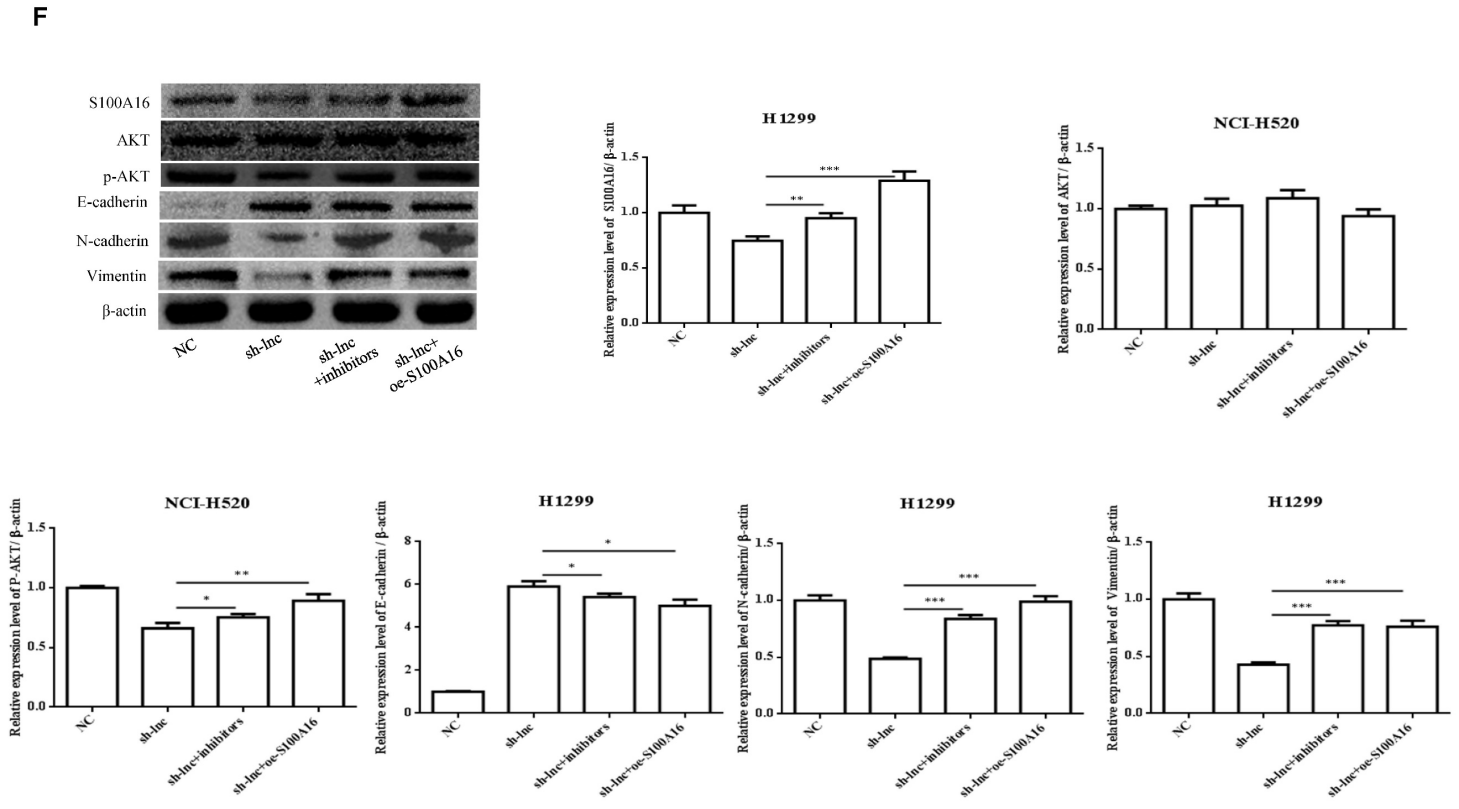
** LncRNA PRKCA-AS1 regulates LUAD progression by modulating S100A16/AKT via sponging miR-508-5p in vitro.** (A) The mRNA expression of PRKCA-AS1, miR-508-5p and S100A16 were assessed by qRT-PCR. (B) CCK-8 assay was used to detect the vitality of NCI-H520 and H1299 cell in different transfected groups. (C) Colony formation experiments was used for proliferation ability of cells in different transfected groups. (D-E) The transwell assay analyzed the cell migration and invasion abilities in cells in different transfected groups. (F) Western blot was used to detect the protein expression of S100A16, AKT, p-AKT, E-cadherin, N-cadherin, and Vimentin. *, *p* < 0.05; **, *p* < 0.01; ***, *p* < 0.001.

To clarify the molecular mechanism of LncRNA PRKCA-AS1 participating in LUAD, we introduced sh-LncRNA PRKCA-AS1 compared with miR-508-5p inhibitor or oe-S100A16 into H1299 cells and then the mRNA expression of PRKCA-AS1, miR-508-5p and S100A16 were assessed by qRT-PCR (Figure 5A). Then, the functional assays of PRKCA-AS1, miR-508-5p and S100A16 on LUAD progression were conducted. Results revealed that knockdown of PRKCA-AS1 restrained the cell proliferation, but it was abolished by miR-508-5p inhibitor or S100A16 overexpression in H1299 cells (Figure 5B-C). Moreover, cell metastasis depressed by PRKCA-AS1 knockdown was induced by decreasing miR-508-5p expression or by oe-S100A16 transfection in H1299 cells through transwell assay (Figure 5D-E). The result of western blot showed that PRKCA-AS1 knockdown could induced the level of E-cadherin but repressed the level of S100A16, p-AKT, N-cadherin, and Vimentin. And co-transfected with miR-508-5p inhibitor or oe-S100A16 could reverse the effect of PRKCA-AS1 knockdown on these proteins (Figure 5F).

### 3.6 LncRNA PRKCA-AS1 regulates LUAD progression by modulating S100A16/AKT via sponging miR-508-5p in vivo

**Figure 6:**
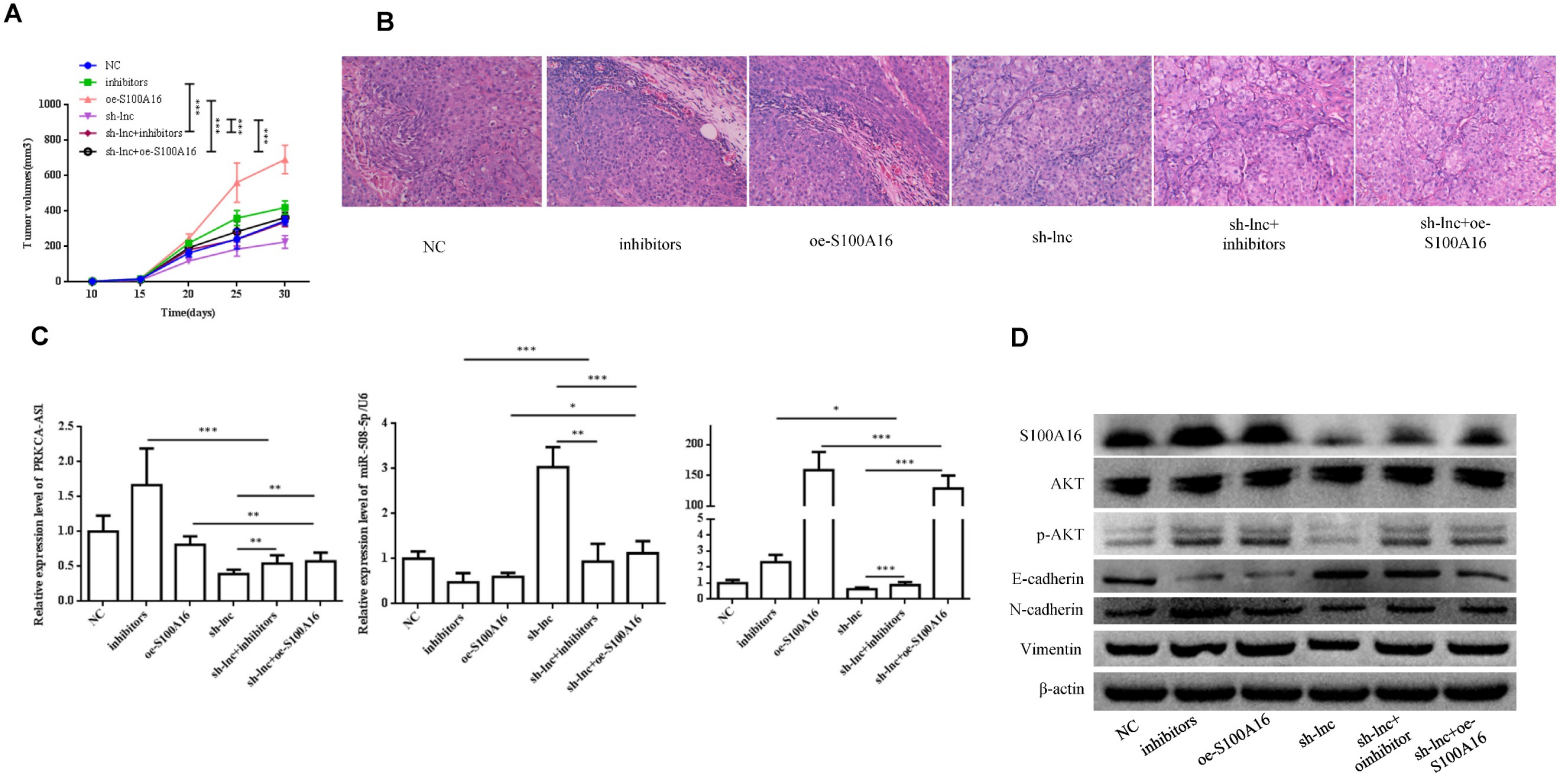

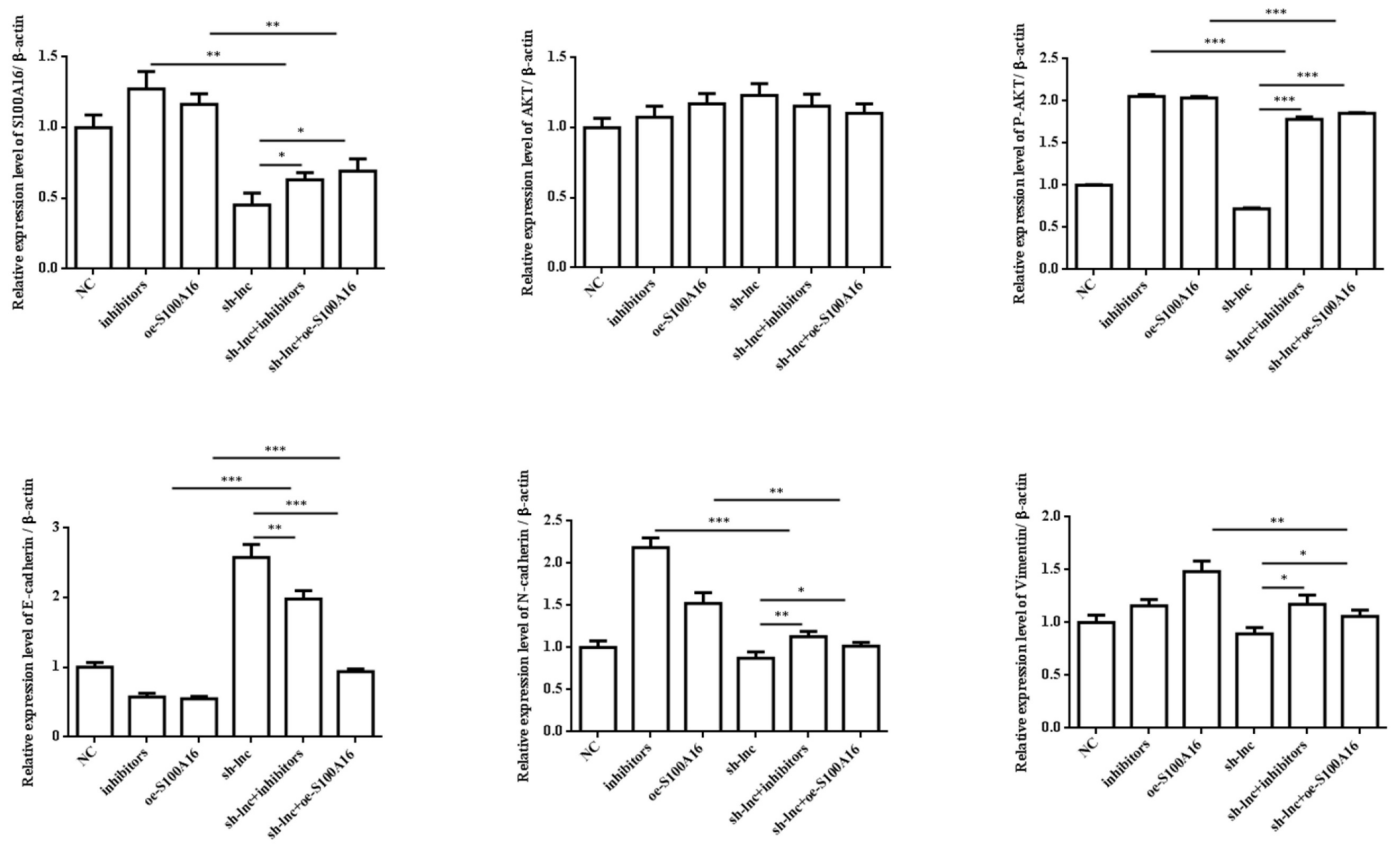
** LncRNA PRKCA-AS1 regulates LUAD progression by modulating S100A16/AKT via sponging miR-508-5p in vivo.** (A) The tumor volume within 30 days after inoculation in mice was analyzed. (B) HE staining experiment was used to observe tumor tissue in mice. (C) The mRNA expression of PRKCA-AS1, miR-508-5p and S100A16 in tumor tissues were assessed by qRT-PCR. (D) Western blot was used to detect the protein expression of S100A16, AKT, p-AKT, E-cadherin, N-cadherin, and Vimentin. *, p < 0.05; **, p < 0.01; ***, p < 0.001.

In vivo experiment, nude mice were randomly assigned into 6 groups (n=8) and inoculated with H1299 cells stablely transfected with NC, inhibitors, oe-S100A16, sh-lnc, sh-lnc+inhibitors and sh-lnc+oe-S100A16. The tumor volume within 30 days after inoculation was analyzed, the group of inhibitors and oe-S100A16 could increase the tumor growth of mice, while sh-lnc group notablely repressed the tumor growth, and there was no significant difference in sh-lnc+inhibitors or sh-lnc+oe-S100A16 group (Figure 6A). HE staining showed that the number of tumor cells increased and some areas showed infiltration and accumulation of inflammatory cells in inhibitors and oe-S100A16 group, while sh-lnc group decreased the tumor cells and the infiltration and accumulation of inflammatory cells (Figure 6B). Additionally, the level of PRKCA-AS1 was increased in the inhibitors group, but it was inhibited in the other groups. The miR-508-5p expression was only increased by sh-lnc group and decreased or had no difference under other groups. And S100A16 could enhanced by oe-S100A16, inhibitors and sh-lnc + oe-S100A16, and it was depressed by sh-lnc (Figure 6C). Furthermore, western blot showed that the expression of related proteins in LUAD tissues of mice in each group was consistent with the results of cell experiments in vitro (Figure 6D). In conclusion, these results suggested that LncRNA PRKCA-AS1 might affect LUAD progression through regulating S100A16/AKT axis via sponge miR-508-5p in vitro and in vivo.

## 4. Discussion

In view of the unsatisfactory survival and high mortality rate of LUAD patients, determining specific and efficient biomarkers for the treatment of LUAD patients has become an urgent concern. Many evidences have confirmed that lncRNA is abnormally expressed in various tumors and has potential application prospects in cancer diagnosis, monitoring, prognosis or prediction of therapeutic response 25. So far, many lncRNAs have been found in LUAD, providing a new perspective for exploring the molecular pathways of LUAD pathogenesis 26. Our study found that PRKCA-AS1 was substantially up-regulated in LUAD tissues and cells, which was related to the adverse clinical results of LUAD patients, so it may be an independent prognostic biomarker of LUAD.

The expression level of PRKCA-AS1 can serve as a significant indicator for assessing the prognosis of LUAD patients. Due to its significant upregulation in tumor tissues, the use of non-invasive biomarker detection methods, such as the detection of free RNA in blood, could facilitate early diagnosis and disease monitoring. Furthermore, the expression level of PRKCA-AS1 may correlate with patients' responses to specific treatment regimens, making it meaningful to consider its potential as a biomarker in personalized treatment strategies. A deeper understanding of the role of PRKCA-AS1 in LUAD will aid in the development of targeted therapeutic approaches. For instance, small molecule inhibitors or RNA interference strategies targeting PRKCA-AS1 could offer new avenues in the treatment of LUAD. Additionally, integrating the expression level of PRKCA-AS1 with other clinical parameters could help in providing more accurate and personalized treatment plans for patients.

CeRNAs are transcripts that can competing with protein-coding mRNAs by binding with shared miRNAs at post-transcription level 27. There was a large amount of evidence summarized the large-scale regulatory network of lncRNA-miRNA-mRNA in cancer cells 28, 29. Some typical "microRNA sponges" such as H19 30, PRKCA-AS1 31, HOTAIR 32 and XIST 33 have been involved in regulating tumor progression in nasopharyngeal carcinoma, uveal melanoma and gastric cancer. In addition, it has been demonstrated that lncRNA MIR99AHG functions as a ceRNA affecting epithelial-mesenchymal transition in LUAD via antagonizing miR-136-5p-mediated USP4 degradation 34. The lncRNA MIR31HG regulates TNFRSF21 by targeting miR-193a-3p, thus acting as an oncogene in LUAD 35. Up to now, the research of LncRNA PRKCA-AS1 in tumor has not been reported, and its potential mechanism is still unknown.

Our present study shows that the increased level of PRKCA-AS1 was apparently leaded to poor survival of LUAD patients. And in vitro experiments showed that overexpression of PRKCA-AS1 notablely promoted cell proliferation and induced metastasis in H1299 or NCI-H520 cells. To elucidate the mechanism of PRKCA-AS1 in LUAD, the target miRNA assumed by PRKCA-AS1 was screened and confirmed. It was found that miR-508-5p was PRKCA-AS1 bound miRNA and was negatively regulated by PRKCA-AS1. Further FISH detection confirmed that PRKCA-AS1 and miR-508-5p were mainly present in the cytoplasm. In addition, miR-508-5p expression was reduced in LUAD tissues, which was consistent with a recent study 36. Also, it has been proved to have anti-tumor effect in endometrial cancer stem cells 37, colorectal cancer 38, lung cancer 39, etc.

S100A16, a member of S100 protein family, has been proved to be up-regulated and cause malignant transformation of various tumors 40, 41. Shang et al. proposed that the aberrant expression of S100A16 might be a prognostic indicator of unfavorable survival in NSCLC 42. S100A16 was proved to participate the adjusting of various signaling pathways, like nuclear factor kappa B pathways 43, extracellular signal regulated kinase 40 and PI3K/AKT 21. Akt, a serine/threonine kinase, plays a key role in PI3K signaling pathway and is often deregulated in diversified human cancers 44. AKT signaling pathway can control kinds of biological activities, including cell proliferation, apoptosis, angiogenesis and metastasis 44, 45. Herein, S100A16 was confirmed as a target of miR-508-5p subsequently, and PRKCA-AS1 overexpression could significantly increase the level of S100A16 and p-AKT, and it was abolished by miR-508-5p mimics in vitro and in vivo. Our study indicated that PRKCA-AS1 might serve as a ceRNA to accelerate S100A16/AKT-mediated LUAD progression by suppressing miR-508-5p.

In our study, we unveiled a novel mechanism by which LncRNA PRKCA-AS1 regulates the expression of S100A16 by sponging miR-508-5p, thereby promoting the progression of LUAD. This discovery not only provides a new perspective for the study of the molecular mechanisms of LUAD but also opens potential avenues for developing novel therapeutic strategies against LUAD.

Given the crucial role of PRKCA-AS1 in LUAD, therapeutic strategies targeting PRKCA-AS1 hold potential clinical value. For example, the development of small molecule inhibitors or the application of RNA interference techniques to target PRKCA-AS1 could effectively inhibit tumor growth and metastasis. Additionally, considering the anti-tumor role of miR-508-5p, enhancing the expression or activity of miR-508-5p could represent another therapeutic approach. This could be achieved through the use of miR-508-5p mimics or by promoting its natural expression. Furthermore, strategies targeting S100A16 also warrant exploration. Given the significant role of S100A16 in the development of LUAD, the development of drugs that can specifically inhibit the activity of S100A16 may benefit LUAD patients. This could include the development of antibody drugs or small molecule inhibitors to block the interaction of S100A16 with its downstream signaling pathways.

In summary, the PRKCA-AS1/miR-508-5p/S100A16 axis not only plays a significant role in the molecular mechanisms of LUAD but also provides potential targets for the development of new therapeutic strategies. Future research should focus on further validating the efficacy of these targets and exploring how these findings can be translated into clinical treatment strategies to improve the prognosis of LUAD patients.

## Supplementary Material

Supplementary table.
