# Supplementary material for: LncRNA PRKCA-AS1 promotes LUAD progression and function as a ceRNA to regulate S100A16 by sponging miR-508-5p
Source: J Cancer. 2024 Jan 27;15(6):1718–30. doi: 10.7150/jca.91184 (PMC10869986; doi:10.7150/jca.91184)
Supplement: Supplementary file 1 — Supplementary table. [file jcav15p1718s1.pdf]

FISH probe	
PRKCA-AS1	5’-DIG-taaaaccaaacggccagatttctaagcg
S100A16	5’-DIG-atatttgtagaagttttccaccaggaca
miR-508-5p	Catgagtgacgccctctggagta
**CHIRP biomarker probes**	
1, Aagtatcaagcttctctggc-bio	
2, Tcttgacatgagcttcaacc-bio	
3, Tttcctgactgactgaaggg-bio	
4, gagtacgacttccaacatgt-bio	
